# A Topographical Atlas of Shiga Toxin 2e Receptor Distribution in the Tissues of Weaned Piglets

**DOI:** 10.3390/toxins8120357

**Published:** 2016-11-30

**Authors:** Daniel Steil, Robert Bonse, Iris Meisen, Gottfried Pohlentz, German Vallejo, Helge Karch, Johannes Müthing

**Affiliations:** 1 Institute for Hygiene, University of Münster, D-48149 Münster, Germany; daniel.steil@ukmuenster.de (D.S.); robert.bonse@gmx.de (R.B.); imeisen@gmx.de (I.M.); pohlentz@uni-muenster.de (G.P.); helge.karch@ukmuenster.de (H.K.); 2 Veterinary practice Dr. med. vet. K. Nolte and Dr. med. vet. G. Vallejo, D-48329 Havixbeck, Germany; g.vallejo@tieraerzte-havixbeck.de

**Keywords:** glycolipids, verotoxin 2e, weaning, Stx2e

## Abstract

Shiga toxin (Stx) 2e of Stx-producing *Escherichia coli* (STEC) is the primary virulence factor in the development of pig edema disease shortly after weaning. Stx2e binds to the globo-series glycosphingolipids (GSLs) globotriaosylceramide (Gb3Cer, Galα1-4Galβ1-4Glcβ1-1Cer) and globotetraosylceramide (Gb4Cer, GalNAcβ1-3Galα1-4Galβ1-4Glcβ1-1Cer), the latter acting as the preferential Stx2e receptor. We determined Stx receptor profiles of 25 different tissues of a male and a female weaned piglet using immunochemical solid phase binding assays combined with mass spectrometry. All probed tissues harbored GSL receptors, ranging from high (category I) over moderate (category II) to low content (category III). Examples of Gb4Cer expression in category I tissues are small intestinal ileum, kidney pelvis and whole blood, followed by colon, small intestinal duodenum and jejunum belonging to category II, and kidney cortex, cerebrum and cerebellum as members of category III organs holding true for both genders. Dominant Gb3Cer and Gb4Cer lipoforms were those with ceramides carrying constant sphingosine (d18:1) and a variable C16:0, C22:0 or C24:1/C24:0 fatty acid. From the mapping data, we created a topographical atlas for Stx2e receptors in piglet tissues and organs, which might be helpful to further investigations on the molecular and cellular mechanisms that underlie infections of Stx2e-producing STEC in pigs and their zoonotic potential for humans.

## 1. Introduction

Among the different Shiga toxin (Stx) subtypes released by Stx-producing *Escherichia coli* (STEC) [1], Stx2e, also known as edema disease verotoxin 2e (VT2e) [2], has been identified as the primary virulence factor involved in the pathogenesis of edema disease in pigs [3,4,5]. In humans, subtypes Stx2a and Stx1a are the most prominent virulence factors of STEC isolates being responsible for severe gastrointestinal diseases and extraintestinal complications, including hemorrhagic colitis and hemolytic uremic syndrome (HUS) [6,7,8]. Cattle are the primary natural reservoir acting as symptomless carriers [9,10,11], although Stx is capable of exerting cytotoxic effects, e.g., on bovine lymphocytes [12,13,14]. In swine, Stx2e-producing STEC cause, typically during the first two weeks after weaning, the edema disease, an enterotoxemia characterized by subcutaneous, mesenteric and cerebral edemas with neurological impairment including ataxia, paralysis, and recumbency as main clinical signs [3]. Stx2e-mediated injury of vascular endothelial cells represents a key event in manifestation of edema disease leading to lesions of focal encephalomalacia in the brain (cerebral softening) with resultant infarction as the main cause of death in affected pigs [3,4]. STEC has emerged as a severe veterinary problem [15,16] and the identification of Stx binding sites in porcine tissues is a key step towards elucidating the mechanisms of Stx-induced cellular damage [17]. Pigs are an ideal model for Stx binding studies because they are susceptible to natural and experimental Stx-mediated disease and develop, e.g., kidney lesions that are comparable to those in humans suffering from HUS [18].

Stxs are built up from an AB_5_ structure consisting of a single A-subunit and five identical B-subunits [19,20,21]. The catalytic A-subunit (“a” stands for “activity”) harbors rRNA *N*-glycosidase activity exerting the cytotoxic effects [22]. The pentameric B-subunit (“b” stands for “binding”) of Stx1a and Stx2a preferentially binds to the cell surface receptor glycosphingolipids (GSLs) globotriaosylceramide (Gb3Cer/CD77) and to a lesser extent to globotetraosylceramide (Gb4Cer) [23,24,25]. On the other hand, Stx2e binds with some preference to Gb4Cer [26], but also recognizes Gb3Cer (the major target of Stx1a and Stx2a) [25,27]. Interestingly, only Stx2e (and neither Stx2a nor Stx1a) binds to Forssman GSL (GalNAcα1-3Gb4Cer) conferring Stx2e a unique recognition feature among the different Stx subtypes analyzed so far [25]. The structures of receptor GSLs and preferential binding of Stx subtypes Stx1a, Stx2a and Stx2e are shown in Figure S1. It is thought that binding of Stxs to clustered GSLs in the outer leaflet of the plasma membrane, subsequent internalization and retrograde routing of the Stx-GSL-complex to intracellular targets are favored by GSL association with microdomains also known as *lipid rafts* [28]. Through multivalent binding to GSLs, Stxs induce lipid clustering and negative membrane curvature, which drives the formation of inward membrane tubules [29,30]. Afterwards, versatile intracellular routes lead the toxins to the lumen of the endoplasmic reticulum, from which they are delivered to the cytosol [20,22,31] or reach the nucleus. This results in host cell protein synthesis inhibition, activation of the ribotoxic stress response, and, in some cases, the induction of apoptosis [32] or damage of the nuclear DNA [33,34].

Gb3Cer and Gb4Cer are characteristic GSLs expressed by human microvascular endothelial cells [35,36,37]. The same Stx receptors have recently been identified in porcine brain capillary endothelial cells [38]. Moreover, we could demonstrate in our previous study Stx2e-mediated strong cytotoxic effects on the endothelial monolayer and rapid collapse of the porcine blood–brain barrier. However, the exact mechanisms of Stx2e-caused cellular damage being involved in the manifestation of the edema disease are only poorly understood. Preliminary studies aimed at the detection of body distribution of Stx2e receptors revealed Stx2e-positive or Stx2e-negative chromatograms for GSL extracts, indicating the presence or absence of globo-series GSLs, respectively, in various tissues and organs [39,40]. Since a comprehensive analysis on the distribution of Stx2e receptors in swine and their possible various lipoforms is missing, we investigated in this study GSL extracts prepared from 25 tissues and organs of a male and a female piglet on their content of the Stx2e receptors Gb3Cer, Gb4Cer and Forssman GSL. For that purpose, we employed thin-layer chromatography (TLC) overlay assays using anti-GSL antibodies and Stx2e, defined the various Stx2e receptor lipoforms by mass spectrometry and performed rank correlation analysis of GSL expression in the two animals. Finally, data are summarized in a topographical atlas of tissues and organs of weaned piglets of both genders.

## 2. Results

GSL-containing lipid extracts were prepared from 25 tissues and organs of a male and a female weaned piglet, both six weeks of age, using small-sized samples of less than 1 g of wet weight (see Table S1) allowing for comprehensive GSL analysis based on highly sensitive technologies combining mass spectrometry with TLC immunostaining. Aliquots of GSL extracts, corresponding to 2 mg of wet weight of respective tissues/organs, were subjected to TLC separation, followed by TLC overlay immunodetection of Stx2e-receptors using anti-Gb3Cer, anti-Gb4Cer, and anti-Forssman GSL antibodies. The structures of the various lipoforms of immunopositive GSLs were then elucidated by means of electrospray ionization (ESI) mass spectrometry (MS), followed by final receptor verification with TLC overlay assays using Stx2e. Hypothesized structures based on MS^1^ analysis were verified by collision-induced dissociation (CID) experiments. An orcinol stain of the reference GSL mixture (R), composed of equimolar concentrations of Gb3Cer, Gb4Cer and Forssman GSL, together with structural formulas of receptor GSLs and an explanation regarding binding preferences of Stx subtypes, are portrayed in Figure S1.

### 2.1. Antibody-Mediated Detection of Gb3Cer in Tissues and Organs of Male Piglet

**Figure 1:**
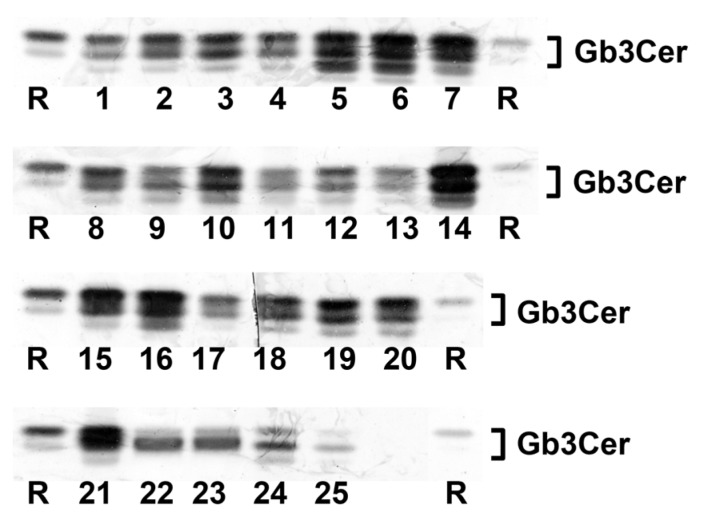
Detection of Stx2e receptors with anti-Gb3Cer antibody in tissues and organs of the male piglet. Aliquots of the GSL extracts of 25 tissues and organs (see Table 1) were applied to TLC separation, followed by TLC overlay assays with polyclonal anti-Gb3Cer antibody. A reference GSL mixture (R) containing equimolar quantities of Gb3Cer, Gb4Cer and Forssman GSL served as positive control. Left-hand and right-hand references correspond to 0.1 µg and 0.02 µg of the GSL mixture, respectively.

Figure 1 shows the TLC overlay detection of Gb3Cer in GSL preparations of the male piglet using the polyclonal anti-Gb3Cer antibody. All tissues/organs were found on first visual inspection to harbor Gb3Cer, but with considerable qualitative and quantitative differences. For instance, the intestinal samples (duodenum (5), jejunum (6), ileum (7), and colon (16), see Figure 1) displayed heavily colored immunopositive triple bands, while Gb3Cer double bands obtained from GSL preparations of the brain (cerebrum (22) and cerebellum (23), see Figure 1) exhibited rather low staining. Because of exact quantitation, immunostained bands were densitometrically analyzed and obtained values were ranked according to their intensities, starting with highest intensity (rank 1), followed by second highest intensity (rank 2), third highest intensity (rank 3), etc. until the sample with the lowest intensity (rank 25) (see Table 1). Based on densitometric ranking, samples were grouped into three categories according to their content of Gb3Cer: category I (high content, rank 1 to 10), category II (moderate or medium content, rank 11 to 19) and category III (low content, rank 20 to 25) (see Table 1). Intestinal samples (duodenum (5), jejunum (6), ileum (7), and colon (16)) belong to category I accompanied by lung (14), spleen (10), small intestinal lymph nodes (15), kidney pelvis (19), urinary bladder (20), and heart (21). The noticeable triple-band patterns of immunostained Gb3Cer suggest remarkable heterogeneity of the ceramide moiety, including variability of fatty acyl chain length and/or postulated hydroxylation of the ceramide lipid anchor. This triple-band pattern was less pronounced in the male samples of moderate content (category II) comprising tissues of nasal bridge (3), eyelid (2), ureter (8), kidney medulla (18), quadriceps muscle (4), liver (12) and the digestive organs stomach (9), pancreas (11) and gall bladder (13). Compared to this, low Gb3Cer content according to category III was characteristic for cerebellum (23) and cerebrum (22), kidney cortex (17), earlobe (1) and whole blood (24, serum plus blood cells). Serum (25) represents the “tissue” with the lowest Gb3Cer content determined in the collection of 25 tissues/organs of the male piglet analyzed.

Most notably, all samples of the intestinal tract belong to category I (high Gb3Cer content), whereas renal samples distribute over the three categories of different concentrations with kidney cortex exhibiting low (category III), kidney medulla showing moderate (category II) and kidney pelvis displaying high content (category I) of Gb3Cer following an increasing receptor gradient of cortex < medulla < pelvis from the outer to the inner part of the organ. Importantly, the organ with almost strongest involvement in the pathogenesis of edema disease, namely the brain with exemplarily analyzed cerebrum and cerebellum, fell into category III according to its very low content of Gb3Cer.

### 2.2. Gb3Cer Lipoforms in Tissues and Organs of Male Piglet

Structures of antibody-detected Gb3Cer in the tissue/organ GSL preparations of the male piglet were identified by ESI mass spectrometry. In a first series of MS experiments we conducted MS^1^ analysis and got Gb3Cer overview mass spectra of positively charged [M + Na]^+^ ions providing data on proposed Gb3Cer structures, which were then further verified by CID analysis of selected precursor ions hereafter referred to as MS^2^.

#### 2.2.1. Gb3Cer Lipoforms in Duodenum and Colon

**Figure 2:**
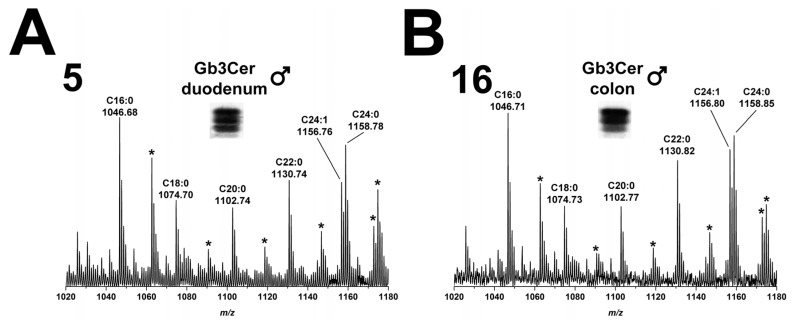
Overview mass spectra of Gb3Cer lipoforms derived from: duodenum (**A**); and colon (**B**) of the male piglet. The MS^1^ spectra display the *m*/*z* range between 1020 and 1180 comprising Gb3Cer lipoforms with variable fatty acid from C16:0 up to C24:1/C24:0 as assigned in the spectrum. All Gb3Cer variants carry constant sphingosine (d18:1) in their respective ceramide moiety. The asterisks point to mass shifts of 15.99 u indicating presence of an additional OH-group in the ceramide moiety, most likely linked to the fatty acyl chain, of Gb3Cer variants in the male: duodenum (**A**); and colon (**B**).

The overview Gb3Cer spectrum of duodenum (5) of the male piglet revealed considerable heterogeneity of detected Gb3Cer variants owing to variability located in the ceramide lipid anchor of the various lipoforms (Figure 2A). Based on the knowledge that all the different Gb3Cer species are characterized by an invariable long-chain aminoalcohol, that is sphingosine (d18:1) in the ceramide moiety (verified later by CID experiments as outlined below), and varying acyl chain length of fatty acids, ions of the MS^1^ spectrum could be assigned to certain Gb3Cer lipoforms. Ions with highest abundance correspond to Gb3Cer variants with C16:0 and C24:1/C24:0 fatty acid at *m*/*z* 1046.68 and 1156.76/1158.78, respectively, accompanied by less abundant ions indicating Gb3Cer species with C18:0, C20:0 and C22:0 fatty acids with *m*/*z* 1074.70, 1102.74, and 1130.74, respectively (Figure 2A). All these ions were flanked by ions with a mass shift increase of 15.99 u and are marked with asterisks in the overview spectrum. This mass shift indicates presence of an additional OH-group in the respective Gb3Cer species. These accompanying ions were preliminarily identified as hydroxylated Gb3Cer variants with Cer (d18:1, C16:0-OH), Cer (d18:1, C18:0-OH), Cer (d18:1, C20:0-OH), Cer (d18:1, C22:0-OH) and Cer (d18:1, C24:1/24:0-OH) as outlined in more detail in the caption of Figure 2A. Collectively, this spectrum provides evidence for highly variable Gb3Cer lipoforms in male duodenum regarding fatty acid chain length and a high extent of hydroxylation of respective fatty acid residues. An example for an experimental CID proof of postulated Gb3Cer lipoforms of duodenum (5) is provided for Gb3Cer (d18:1, C16:0-OH) in Figure S2, which shows verification of the proposed structure of the hydroxylated Gb3Cer variant as deduced from the fragment ions obtained by CID of the precursor ion at *m/z* 1062.65. The same repertoire of ions was detected in the GSL preparation obtained from colon (16) of the male piglet as shown in Figure 2B. Thus, colon is almost identical with regard to Gb3Cer expression when compared to intestinal duodenum and the spectrum in Figure 2B does not require additional explanations.

#### 2.2.2. Gb3Cer Lipoforms in Kidney Cortex, Medulla, and Pelvis

**Figure 3:**
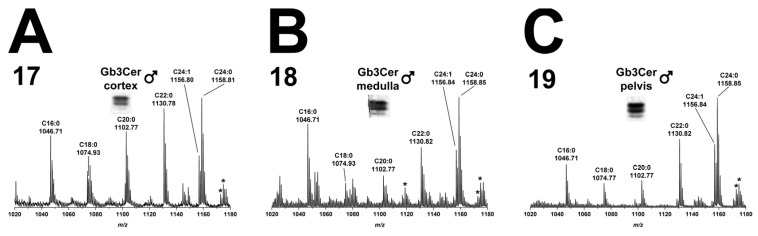
Overview mass spectra of Gb3Cer lipoforms derived from: cortex (**A**); medulla (**B**); and pelvis (**C**) of the male piglet. The MS^1^ spectra depict the *m*/*z* range from 1020 to 1180 encompassing Gb3Cer variants with variable fatty acid from C16:0 up to C24:1/C24:0 as marked in the spectrum. All Gb3Cer variants harbor constant sphingosine (d18:1) in their respective ceramide moiety. The asterisks indicate hydroxylation of the ceramide portion, most likely of the fatty acid, of Gb3Cer variants in the male: cortex (**A**); medulla (**B**); and pelvis (**C**).

The overview mass spectra, which were obtained by ESI mass spectrometry from the GSL extracts of immunostained Gb3Cer bands derived from kidney cortex (17), kidney medulla (18), and kidney pelvis (19) are shown in Figure 3A–C, respectively. In cortex, the ladder of ions with increasing *m*/*z* values starting from Gb3Cer (d18:1, C16:0) at *m*/*z* 1046.71 up to Gb3Cer (d18:1, C24:1/C24:0) at *m*/*z* 1156.80/1158.81 (Figure 3A), indicates the presence of the same Gb3Cer variants as detected in duodenum and colon of the male piglet with invariable sphingosine (d18:1) and variable fatty acids with C16:0, C18:0, C20:0, C22:0 and C24:1/C24:0 acyl chains. The extent of ceramide hydroxylation was minimal in the kidney and the only hydroxylated Gb3Cer species, which appeared as very low abundant ions by a mass shift of 15.99 u in the MS^1^ spectra (marked with asterisks), were Gb3Cer (d18:1, C24:1/C24:0-OH) in cortex (17, Figure 3A), medulla (18, Figure 3B) and pelvis (19, Figure 3C) and Gb3Cer (d18:1, C20:0-OH) in medulla (18, Figure 3B). The experimental CID proof of proposed Gb3Cer (d18:1, C24:0-OH) of medulla (17) is given in Figure S3, which shows CID proof of the postulated Gb3Cer. Collectively, overview spectra indicated mostly uniform expression of individual Gb3Cer species in kidney cortex, medulla and pelvis, although quantitative differences were considerably high in the different areas of this organ (see Figure 1 and Table 1).

### 2.3. Antibody-Mediated Detection of Gb4Cer in Tissues and Organs of Male Piglet

**Figure 4:**
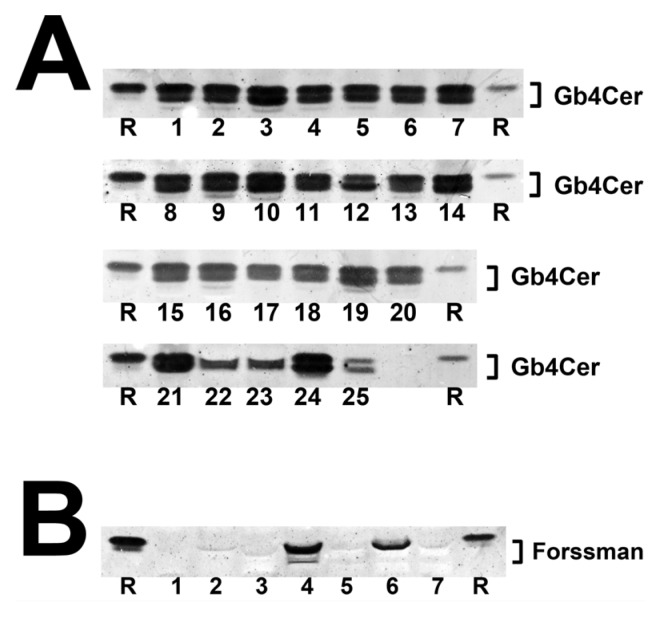
Detection of Stx2e receptors with: anti-Gb4Cer (**A**); and anti-Forssman GSL antibody (**B**) in tissues and organs of the male piglet. Aliquots of the GSL extracts of 25 tissues and organs (see Table 1) were applied to TLC separation, followed by TLC overlay assays with: polyclonal anti-Gb4Cer antibody (**A**); or monoclonal anti-Forssman GSL antibody (**B**). A reference GSL mixture (R) containing equimolar quantities of Gb3Cer, Gb4Cer and Forssman GSL was used as positive control. References on the left and the right correspond: to 0.1 µg and 0.02 µg of the GSL mixture, respectively, for the anti-Gb4Cer assay (**A**); and to 0.015 µg and 0.003 µg of the GSL mixture, respectively, for anti-Forssman GSL detection (**B**).

The TLC overlay detection of Gb4Cer, which represents the preferential GSL receptor of Stx2e (see explanations in Figure S1), with polyclonal anti-Gb4Cer antibody is shown for GSL preparations of the male piglet in Figure 4A. Facing the TLC overlay chromatograms it becomes obvious that all tissues/organs contain considerable but somewhat varying amounts of Gb4Cer. The immunostained bands were scrutinized densitometrically, ranks were conferred on measurements and tissues/organs were assigned (as before done with Gb3Cer bands) to category I, category II or category III (see Table 1). For example, ileum (7) was found to belong to category I, together with other organs such as spleen (10), kidney pelvis (19), lung (14), pancreas (11), and stomach (9) and tissues of nasal bridge (3), earlobe (1), and ureter (8) as well as whole blood (24). Other intestinal samples (duodenum (5), jejunum (6), and colon (16)) belong to category II accompanied, e.g., by heart (21) and gall bladder (13). Remarkably, Gb4Cer expression in kidney tissue (pelvis (19), medulla (18), and cortex (17)) was associated with category I (high Gb4Cer content), category II (moderate Gb4Cer content), and category III (low Gb4Cer content), respectively, indicating same grouping as determined for Gb3Cer (see above). Again, cerebellum (23) and cerebrum (22) ranged in category III, indicating that the brain contained only very low amounts of Gb4Cer. Collectively, for the most part the Gb4Cer distribution resembles that one of Gb3Cer but does not really correlate with Gb3Cer expression. The calculated rank correlation coefficient ρ = 0.372 (*p* = 0.0676) indicates rather low association between Gb3Cer and Gb4Cer expression in the male piglet. The double-band patterns being characteristic for all Gb4Cer bands suggest less pronounced ceramide variability of Gb4Cer when compared to Gb3Cer with regard to a negligible degree of fatty acid hydroxylation.

### 2.4. Antibody-Mediated Detection of Forssman GSL in Tissues and Organs of Male Piglet

The Forssman GSL represents an αGalNAc-extended globoside (Gb4Cer) structure (α3GalNAc-Gb4Cer). Among the various Stx subtypes analyzed so far in detail (Stx1a, Stx2a and Stx2e), Stx2e is the only one, which binds to this pentahexosylceramide (Figure S1). We employed a monoclonal anti-Forssman antibody being capable to detect this GSL in low nanogram quantities in GSL extracts [25,41]. From all 25 samples analyzed, Forssman GSL could be detected only in TLC overlay assay chromatograms of quadriceps muscle (4) and jejunum (6) as shown in Figure 4B, whereas all other tissues/organs were negative in TLC binding assays. Because of the extremely low concentrations of Forssman GSL, structural characterization by ESI mass spectrometry could not be performed.

### 2.5. Gb4Cer Lipoforms in Tissues and Organs of Male Piglet

As described above for antibody-detected Gb3Cer, Gb4Cer variants detectable with anti-Gb4Cer antibody in the GSL preparations of the 25 tissues/organs of the male piglet (see Figure 4A) were structurally characterized by ESI mass spectrometry using the positive ion mode.

#### 2.5.1. Gb4Cer Lipoforms of Duodenum and Colon

**Figure 5:**
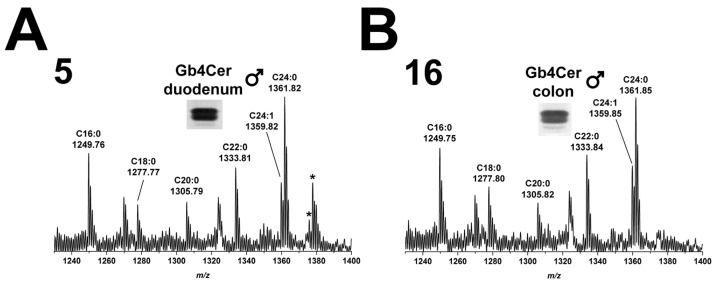
Overview mass spectra of Gb4Cer lipoforms derived from: duodenum (**A**); and colon (**B**) of the male piglet. The MS^1^ spectra display the *m*/*z* range between 1230 and 1400 comprising Gb4Cer lipoforms with variable fatty acid from C16:0 up to C24:1/C24:0 as assigned in the spectrum. All Gb4Cer variants carry constant sphingosine (d18:1) in their respective ceramide moiety. The two asterisks point to hydroxylated Gb4Cer in the male duodenum (**A**), whereas no hydroxylated Gb4Cer lipoforms were detectable in the male colon (**B**).

MS^1^ analysis of Gb4Cer extracted from duodenum (5) of the male piglet and identified with anti-Gb4Cer antibody revealed occurrence of several Gb4Cer variants as shown in the overview spectrum of Figure 5A. Proposed structures are Gb4Cer lipoforms with sphingosine (d18:1) and fatty acids with increasing chain length from C16:0 to C24:1/C24:0 as highlighted in the spectrum. The prevalent species were Gb4Cer (d18:1, C16:0) and Gb4Cer (d18:1, C24:1/C24:0), the latter accompanied by less abundant ions indicating presence of Gb4Cer with hydroxylated C24:1/C24:0 fatty acids (shift of 15.99 u). These structures were also found in the MS^1^ spectrum obtained from the GSL extract of colon (16) of the male piglet (Figure 5B) indicating the presence of identical Gb4Cer variants in both intestinal samples with the exception of minor signals corresponding to Gb4Cer (d18:1, C24:1/C24:0-OH) in duodenum (5), which are absent in colon (16).

#### 2.5.2. Gb4Cer Lipoforms in Kidney Cortex, Medulla, and Pelvis

**Figure 6:**
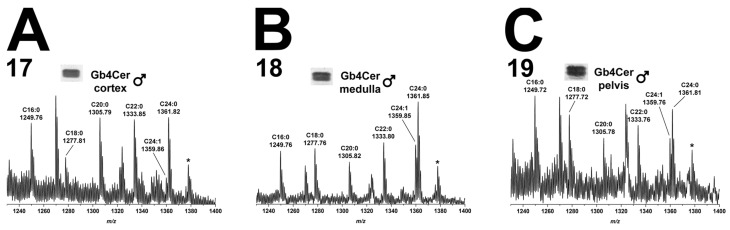
Overview mass spectra of Gb4Cer lipoforms derived from: cortex (**A**); medulla (**B**); and pelvis (**C**) of the male piglet. The MS^1^ spectra depict the *m*/*z* range from 1230 to 1400 encompassing Gb4Cer variants with variable fatty acid from C16:0 up to C24:1/C24:0 as marked in the spectrum. All Gb4Cer variants harbor constant sphingosine (d18:1) in their respective ceramide moiety. The single asterisk in each spectrum indicates hydroxylated Gb4Cer in the male: cortex (**A**); medulla (**B**); and pelvis (**C**).

The data of mass spectra obtained from the GSL extracts of kidney cortex (17), medulla (18), and pelvis (19) are shown in Figure 6. Gb4Cer species carrying sphingosine (d18:1) being linked with a fatty acid ranging from C16 to C24 occurred in the three kidney regions of male piglet. The extent of hydroxylation was marginal, since only one hydroxylated species, Gb4Cer (d18:1, C24:0-OH), appeared as low abundant ions in the three spectra (Figure 6).

### 2.6. Stx2e-Mediated Detection of Stx2e Receptors Gb3Cer and Gb4Cer in Tissues and Organs of Male Piglet

GSL receptors of Stx2e have been detected so far by use of polyclonal anti-Gb3Cer and anti-Gb4Cer antibodies as well as monoclonal anti-Forssman GSL antibody. The advantage of using antibodies lies in the fact that they can detect even trace quantities of GSLs in complex GSL mixtures as outlined in previous publications [38,42,43,44]. Although being considerably less sensitive in receptor detection, we employed Stx2e in combination with anti-Stx2 antibody in addition to the antibody-mediated detection of Stx2e receptors. The result of Stx2e TLC overlay assays should be a merged picture of anti-Gb3Cer (Figure 1) and anti-Gb4Cer staining (Figure 4A). Forssman GSL is absent in 23 tissue/organs of male piglet and present in only trace quantities in two tissues (Figure 4B).

**Figure 7:**
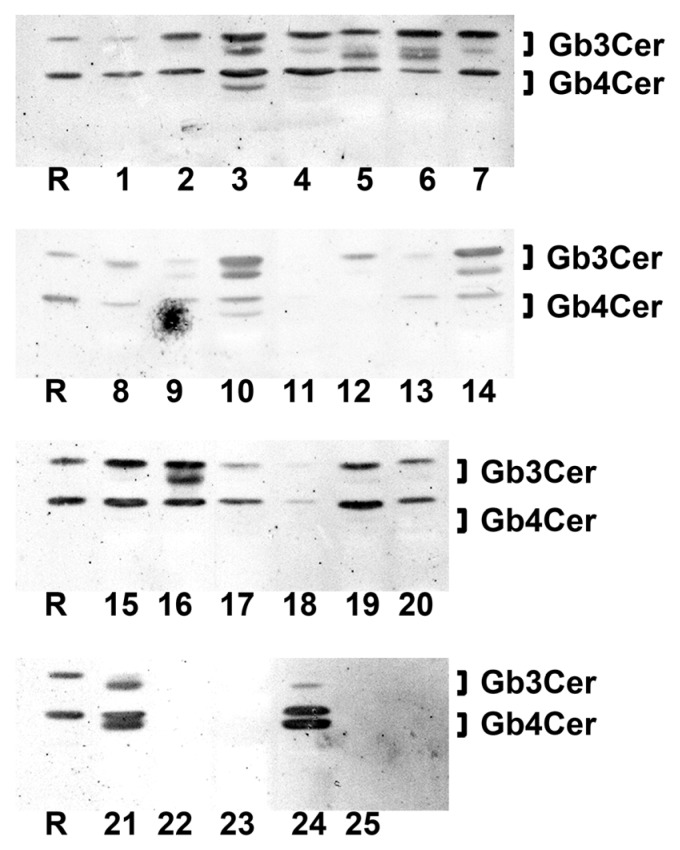
Detection of receptor GSLs with Stx2e in tissues and organs of the male piglet. Aliquots of the GSL extracts of 25 tissues and organs (see Table 1) were applied to TLC separation, followed by TLC overlay assays with Stx2e. A reference GSL mixture (R) containing equimolar quantities of Gb3Cer, Gb4Cer and Forssman GSL served as positive control (0.1 µg).

As shown in Figure 7, clear Stx2e-positive doublets are visible in case of nasal bridge (3), spleen (10), lung (14), colon (16) and less pronounced in duodenum (5) and jejunum (6), whereas a very faint double band was seen in case of stomach (9). In GSL extracts of eyelid (2), quadriceps muscle (4), ileum (7), intestinal lymph nodes (15), kidney pelvis (19) and heart (21) strong Stx2e-positive upper Gb3Cer bands (harboring the Gb3Cer species with long chain C22–C24 fatty acids) could be detected. A faint positive Gb3Cer upper band was visible in the samples of earlobe (1), ureter (8), liver (12), kidney cortex (17) and whole blood (24) (Figure 7). The remaining seven tissues/organs were negative regarding Gb3Cer recognition of Stx2e (most likely due to low content of this GSL in the respective extract) including cerebrum (22) and cerebellum (23).

In addition to the interaction of Stx2e with Gb3Cer, Figure 7 displays Stx2e-mediated recognition of Gb4Cer, the favored GSL receptor of Stx2e. Double bands with extremely high intensity were detected in GSL preparations of whole blood (24) and heart (21) as well as a moderately intensive doublet in case of nasal bridge (3) and a weakly stained doublet in spleen (10). Strong positive single Stx2e-positive Gb4Cer upper bands could be determined in GSL extracts of intestinal lymph nodes (15), colon (16) and kidney pelvis (19), followed by moderately stained single Gb4Cer bands in eyelid (2), quadriceps muscle (4), ileum (7), kidney cortex (17) and urinary bladder (20). Slightly positive reactions were observed for earlobe (1), duodenum (5) and jejunum (6), traces for ureter (8), stomach (9), gall bladder (13), lung (14) and kidney medulla (18). Negative results were obtained for pancreas (11), liver (12), cerebrum (22), cerebellum (23) and serum (25). In summary, it is noteworthy that the Stx2e binding assays correspond to the anti-Gb3Cer and anti-Gb4Cer antibody assays. Importantly, the sample of whole blood exhibited the strongest interaction with Stx2e being in agreement with the high content (but not highest among the analyzed tissue/organs) of Gb4Cer (see Table 1), which might have functional impact on the putative role of blood cells as possible transport vehicles of Stx2e through the circulation of infected animals.

### 2.7. Antibody-Mediated Detection of Gb3Cer in Tissues and Organs of Female Piglet

**Figure 8:**
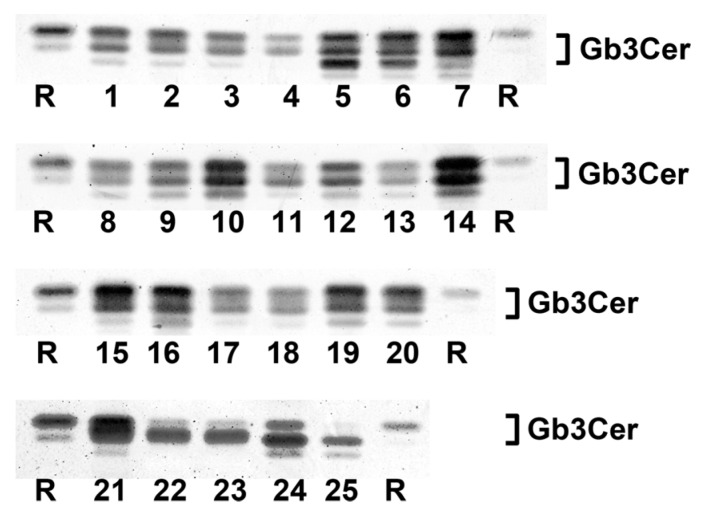
Detection of Stx2e receptors with anti-Gb3Cer antibody in tissues and organs of the female piglet. Aliquots of the GSL extracts of 25 tissues and organs (see Table 1) were applied to TLC separation, followed by TLC overlay assays with polyclonal anti-Gb3Cer antibody. A reference GSL mixture (R) containing equimolar quantities of Gb3Cer, Gb4Cer and Forssman GSL served as positive control. Left-hand and right-hand references correspond to 0.1 µg and 0.02 µg of the GSL mixture, respectively.

TLC overlay detection of Gb3Cer in GSL extracts derived from the weaned female piglet using polyclonal anti-Gb3Cer antibody is displayed in Figure 8.

All tissues/organs of the female animal were positive in terms of Gb3Cer expression and exhibited sizeable qualitative and quantitative differences being very similar to those observed in the male piglet. In analogy to the male animal and giving some examples, kidney pelvis (19) and intestinal samples (duodenum (5), jejunum (6), ileum (7), and colon (16)) were found to rank among the samples of category I due to their high Gb3Cer content, followed by kidney cortex (17) and kidney medulla (18) with moderate Gb3Cer amounts in category II and cerebrum (22) and cerebellum (23) almost at the end of the ranking coming under category III (Table 1). TLC overlay ranks of Gb3Cer detection in tissues/organs of the female piglet correlated with those of the male piglets (ρ = 0.797, *p* = 3.9 × 10^−6^) determined on 1% significance level indicating strong association of Gb3Cer expression between the male and the female piglet. Thus, the almost largely consistent results suggest gender independent Gb3Cer expression in weaned piglets of six weeks of age.

### 2.8. Gb3Cer Lipoforms in Tissues and Organs of Female Piglet

Positive ion mode ESI mass spectrometry allowed for identification of the various Gb3Cer lipoforms detected in the GSL extracts of immunopositive Gb3Cer bands obtained from tissue/organ samples of the female piglet (see Figure 8). Proposed structures deduced from MS^1^ spectra were afterwards confirmed by CID measurements.

#### 2.8.1. Gb3Cer Lipoforms in Duodenum and Colon

Basically, the same Gb3Cer lipoforms were detected when compared to the male piglet regarding Gb3Cer variants with ceramides composed of sphingosine (d18:1) and C16:0, C18:0, C20:0, C22:0 or C24:1/C24:0 fatty acid residues with clear dominance of Gb3Cer (d18:1, C16:0) and Gb3Cer (d18:1, C24:1/C24:0) in most of the spectra. Two examples of overview mass spectra for Gb3Cer of duodenum (5) and colon (16) of the female animal are provided in Figure S4. In some cases the spectra of the female piglet showed somewhat higher background noise, but identification of Gb3Cer molecules was not compromised by this means (see Figure S4). An evident difference in comparison to the male animal was the diminished extent of hydroxylation of Gb3Cer signified by absence of GSL species with C18:0-OH, C20:0-OH and C22:0-OH fatty acids, which were detectable in the spectra of the male animal in duodenum (5) and colon (16) (see Figure 2A,B, respectively). However, it should be straightened out that this finding is restricted to the intestinal samples (where this difference was observed) and does not contradict the principal correlation of Gb3Cer expression in the male and the female piglet.

#### 2.8.2. Gb3Cer Lipoforms in Kidney Cortex, Medulla, and Pelvis

Since the spectra obtained from female kidney are identical to those of the male piglet, the overview mass spectra of Gb3Cer of kidney cortex (17), kidney medulla (18) and kidney pelvis (19) of the female animal are depicted in Figure S5. The same pattern of non-hydroxylated Gb3Cer variants with Cer (d18:1, C16:0) up to Cer (d18:1, C24:1/C24:0) with minimal hydroxylation in form of Gb3Cer (d18:1, C24:1/C24:0-OH) was identified as before in the three corresponding spectra of the male piglet (see Figure 3A–C, respectively).

### 2.9. Antibody-Mediated Detection of Gb4Cer in Tissues and Organs of Female Piglet

**Figure 9:**
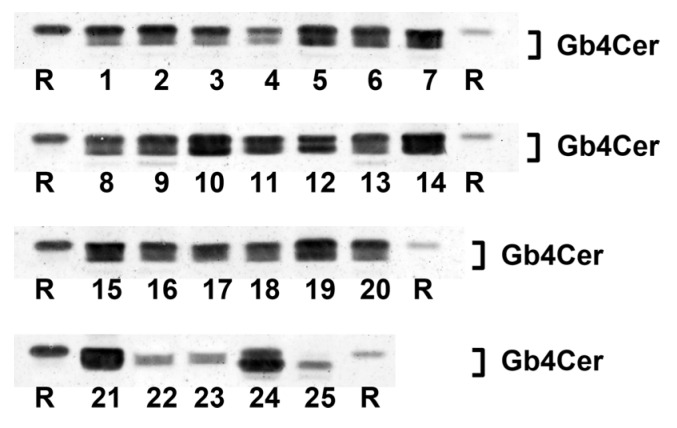
Detection of Stx2e receptors with anti-Gb4Cer antibody in tissues and organs of the female piglet. Aliquots of the GSL extracts of 25 tissues and organs (see Table 1) were applied to TLC separation, followed by TLC overlay assays with polyclonal anti-Gb4Cer antibody. A reference GSL mixture (R) containing equimolar quantities of Gb3Cer, Gb4Cer and Forssman GSL was used as positive control. References on the left and the right correspond to 0.1 µg and 0.02 µg of the GSL mixture, respectively.

Figure 9 shows TLC overlay detection of Gb4Cer (the preferred Stx2e receptor as explained in Figure S1) in tissue/organs of the female piglet using the polyclonal anti-Gb4Cer antibody. Obviously most of the tissues/organs exhibit strongly stained Gb4Cer-positive bands giving evidence for ubiquitous presence of Gb4Cer in all analyzed samples. GSL extracts of spleen (10) and lung (14) occupy the two top rank positions accompanied by kidney pelvis (19) in the middle and ileum (7) at the bottom part of category I (high content, rank 1 to 10) scaled samples (see Table 1). The duodenum (5) and jejunum (6) range in the upper area of sample ranking under category II (moderate content, rank 11 to 19) followed by colon (16), which occupies a mid-table position of this category. The kidney medulla (18) and kidney cortex (17) values distribute to category III (low content, rank 20 to 25) and cerebellum (23) and cerebrum (22) ranked at the end-third and bottom position of the table. As found for the male animal, again cerebellum (23) and cerebrum (22) ranged in category III, indicating that also the brain of the female piglet contained only very low amounts of Gb4Cer. In case of the female animal, the Gb4Cer ranking correlated with the rank order of its Gb3Cer contents, evidenced by a calculated rank correlation coefficient of ρ = 0.656 (*p* = 5 × 10^−4^) determined on 1% significance level. The same (that is association) holds true when comparing Gb4Cer expression in the male and the female piglet, indicated by a rank correlation coefficient of ρ = 0.69 (*p* = 2 × 10^−4^) calculated on 1% significance level. The uniform double-band patterns suggest very little extent of fatty acid hydroxylation of Gb4Cer molecules in the tissues/organs of the female piglet as proved by MS^1^ and MS^2^ analyses and demonstrated in the following paragraph.

The Forssman GSL could not be detected in any of the 25 tissues/organs of the female piglet despite the fact that an extremely sensitive monoclonal anti-Forssman GSL antibody was employed. The negative results of the TLC overlay approaches are therefore not shown.

### 2.10. Gb4Cer Lipoforms in Tissues and Organs of Female Piglet

Because Gb4Cer acts as the preferred GSL receptor for Stx2e among the globo-series neutral GSLs, the Gb4Cer ESI MS^1^ spectra obtained in positive ion mode from anti-Gb4Cer positive TLC overlay bands of the female piglet (see Figure 9), although being very similar to those received from the tissues/organs of the male piglet, are shown for two intestinal and the three kidney samples in this paragraph. Using CID investigation, the anticipated structures were approved upon MS^1^ analysis.

#### 2.10.1. Gb4Cer Lipoforms of Duodenum and Colon

**Figure 10:**
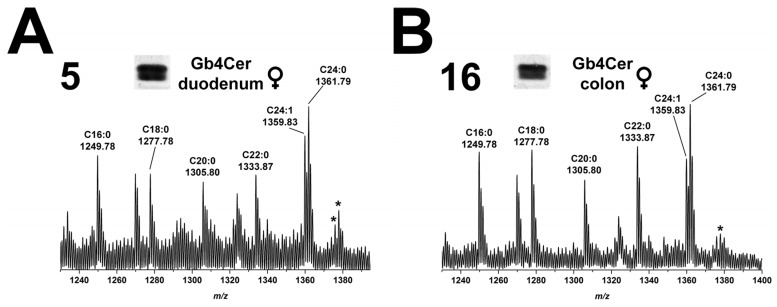
Overview mass spectra of Gb4Cer lipoforms derived from: duodenum (**A**); and colon (**B**) of the female piglet. The MS^1^ spectra display the *m*/*z* range between 1230 and 1400 comprising Gb4Cer lipoforms with variable fatty acid from C16:0 up to C24:1/C24:0 as assigned in the spectrum. All Gb4Cer variants carry constant sphingosine (d18:1) in their respective ceramide moiety. The asterisks indicate hydroxylated Gb4Cer in the female: duodenum (**A**); and colon (**B**).

Detected Gb4Cer molecules in duodenum (5) and colon (16) of the female piglet (Figure 10A,B, respectively) are the ubiquitously found species with ceramides harboring sphingosine (d18:1) coupled with a fatty acid from C16 up to C24 chain length, whereby the latter is accompanied by low abundant hydroxylated ions (shift by 15.99 u). These ions were the same compared to those obtained from the male piglet (see Figure 5) indicating an extremely high degree of homology of Gb4Cer expression in the tissues/organs of both genders.

#### 2.10.2. Gb4Cer Lipoforms in Kidney Cortex, Medulla, and Pelvis

**Figure 11:**
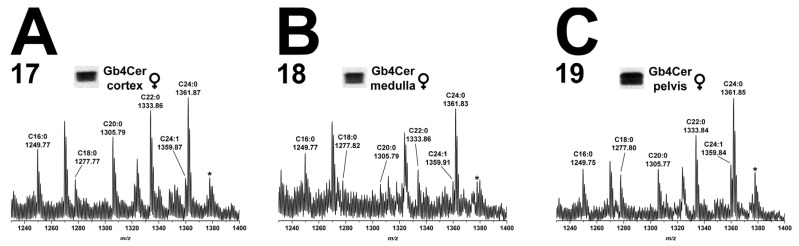
Overview mass spectra of Gb4Cer lipoforms derived from: cortex (**A**); medulla (**B**); and pelvis (**C**) of the female piglet. The MS^1^ spectra depict the *m/z* range from 1230 to 1400 encompassing Gb4Cer variants with variable fatty acid from C16:0 up to C24:1/C24:0 as marked in the spectrum. All Gb4Cer variants harbor constant sphingosine (d18:1) in their respective ceramide moiety. The single asterisk in each spectrum indicates hydroxylated Gb4Cer in the female: cortex (**A**); medulla (**B**); and pelvis (**C**).

The overview mass spectra of the Gb4Cer molecules attained from the kidney cortex (17), kidney medulla (18), and kidney pelvis (19) are depicted in Figure 11A–C, respectively. The spectra widely correspond to the ones which were obtained from the male kidney (see Figure 6), and a detailed description becomes therefore superfluous in terms of avoiding reiterating interpretation.

### 2.11. Stx2e-Mediated Detection of Stx2e Receptors Gb3Cer and Gb4Cer in Tissues and Organs of Female Piglet

**Figure 12:**
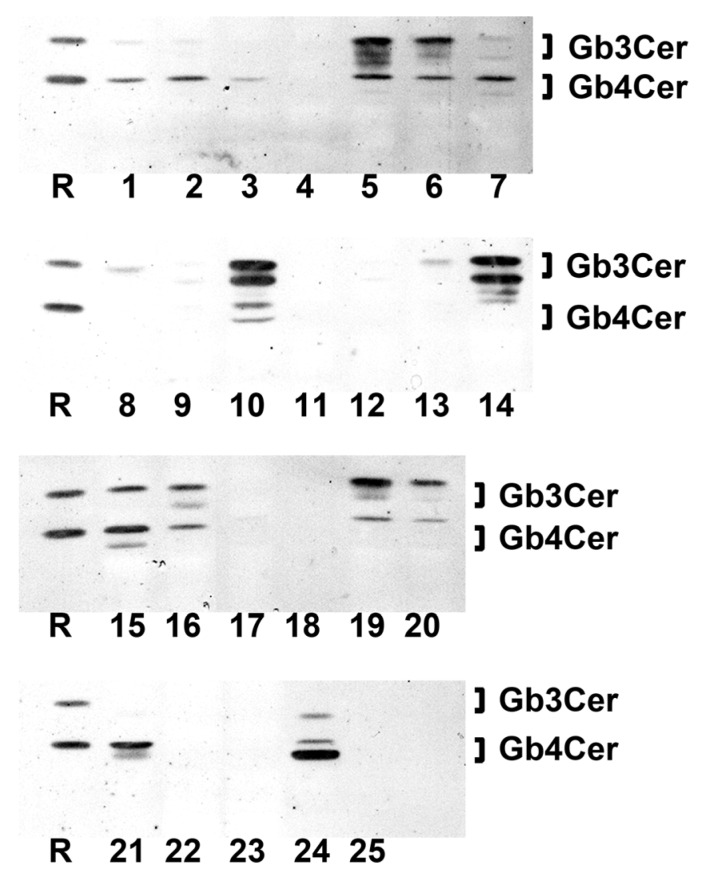
Detection of receptor GSLs with Stx2e in tissues and organs of the female piglet. Aliquots of the GSL extracts of 25 tissues and organs (see Table 1), were applied to TLC separation, followed by TLC overlay assays with Stx2e. A reference GSL mixture (R) containing equimolar quantities of Gb3Cer, Gb4Cer and Forssman GSL served as positive control (0.1 µg).

The employment of Stx2e, combined with anti-Stx2 antibody, resulted in Stx2e binding patterns indicating recognition of both Gb3Cer and Gb4Cer as shown in Figure 12 and as observed before for the GSL extracts derived from organs of the male piglet (see Figure 7). With respect to Gb3Cer detection, particularly eye-catching are the vigorously stained Gb3Cer doublets in the spleen (10) and the lung sample (14) and the heavily stained Gb3Cer triplet detected in the GSL extract of duodenum (5). Slightly colored double bands were observed for jejunum (6) and colon (16) and kidney pelvis (19), followed by less intensive single Gb3Cer bands for small intestinal lymph nodes (15), urinary bladder (20) and whole blood (24) as well as traces in earlobe (1), eyelid (2), ileum (7), ureter (8), and gall bladder (13). The remnant eleven samples were negative in the Stx2e overlay assay regarding Gb3Cer detection.

Stx2e-mediated interaction with Gb4Cer resulted in double bands in the GSL extracts of spleen (10), lung (14), small intestinal lymph nodes (15), heart (21) and whole blood (24), whereof highest intensity was observed for the whole blood sample with disproportional strong binding towards the lower band of the Gb4Cer doublet. Opposite binding of Stx2e was visible in case of small intestinal lymph nodes (15) and heart (21) with respect to higher upper band intensities compared to the lower ones. Gb4Cer was further detected as a moderately colored single band in earlobe (1), eyelid (2), duodenum (5), jejunum (6), ileum (7), colon (16) and kidney pelvis (19), whereas only a poor positive reaction was recognizable for nasal bridge (3) and urinary bladder (20). Gb4Cer was undetectable with the toxin in the other nine tissues/organs, remarkably in kidney cortex (17) and kidney medulla (18) and the brain samples of cerebrum (22) and cerebellum (23). Notably, strongest interaction was detectable for Stx2e in whole blood (24) as observed before in the male piglet (see Figure 7). All in all, the Stx2e receptor profiles were on the one hand similar to those obtained from male animal, but exhibited on the other hand less heavily stained Stx2e receptors when compared to stronger staining in the male animal, or gave negative results in comparison to weakly positive tissue/organ extracts of the male piglet. Taken together, it therefore seems that the female animal expressed lower contents of Gb3Cer and Gb4Cer, a fact that became disclosed when using Stx2e in TLC overlay assays.

### 2.12. Compiled Distribution of Antibody-Detected Stx2e Receptor Gb3Cer in Tissues and Organs of Male and Female Piglet

**Figure 13:**
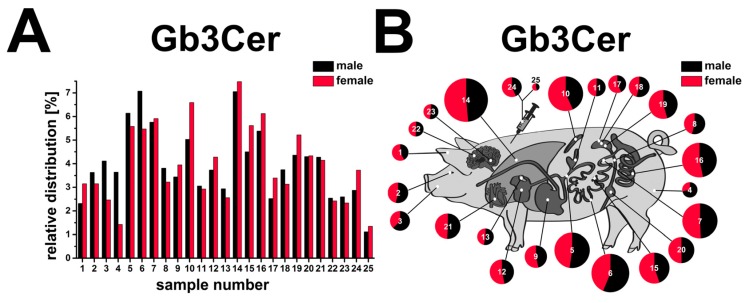
Relative content of antibody-detected Stx2e receptor Gb3Cer in tissues and organs of male and female piglet. Gb3Cer distribution to the various tissues and organs of the male and the female piglet is depicted as a bar chart (**A**) in the order of the sample numbers from left to right according to Figure 1 (male piglet) and Figure 8 (female piglet) and as a schematic topographical atlas in the form of a cartoon map of pig tissues/organs (**B**). The size of the circle diameters corresponds to the relative Gb3Cer amount and the scale of the “pie slices” indicates the relative Gb3Cer content of the male (black) and the female animal (red).

The bar chart presented in Figure 13A portrays the relative percentages of Gb3Cer in direct comparison of the male (black) and the female (red) tissues/organs. The chart illustrates the remarkable differences of Gb3Cer in the various tissues/organs of each animal. Peak values of 7.1% for jejunum (6) and 7.1% for lung (14) represent the Gb3Cer expression maxima for the male piglet, followed by 6.1% of duodenum (5), 5.8% of ileum (7), and 5.4% of colon (16) representing the ≥5% group of tissues/organs. On the other hand, male tissues/organs harboring relative Gb3Cer amounts of ≤2.5% (less than half or even less than half of the frontrunners) are cerebrum (22) with 2.5% and kidney cortex (17) with 2.5%, followed by earlobe (1) with 2.3% and bottom-placed serum (25) with 1.1%. Thus, sixteen of the twenty-five sample values ranged in the interval between ≥2.5% and ≤5%. The animal cartoon (Figure 13B) visualizes the tissue/organ distribution of the two piglets as a topographical atlas, whereby sizes of the circle diameters and the “cake pieces” correspond to the relative Gb3Cer quantities. The same trends could be verified for relative Gb3Cer contents in tissues/organs of the female piglet and are not further outlined.

### 2.13. Compiled Distribution of Antibody-Detected Stx2e Receptor Gb4Cer in Tissues and Organs of Male and Female Piglet

**Figure 14:**
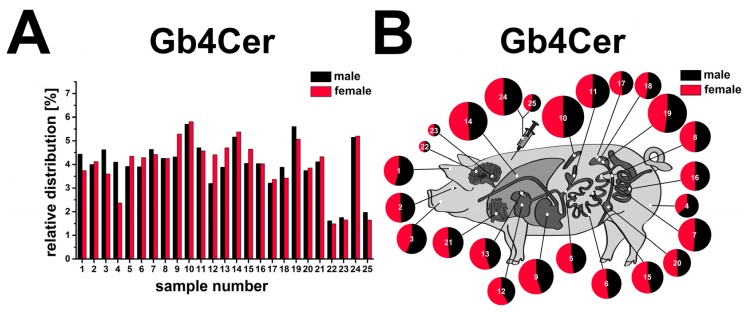
Relative content of antibody-detected Stx2e receptor Gb4Cer in tissues and organs of male and female piglet. Gb4Cer distribution to the various tissues and organs of the male and the female piglet is depicted as a bar chart (**A**) in the order of the sample numbers from left to right according to Figure 4A (male piglet) and Figure 9 (female piglet) and as a schematic topographical atlas in the form of a cartoon map of pig tissues/organs (**B**). The size of the circle diameters corresponds to the relative Gb4Cer amount and the scale of the “pie slices” indicates the relative Gb4Cer content of the male (black) and the female animal (red).

The relative percentages of Gb4Cer contents attained from the tissues/organs of the male (black) and the female animal (red) are directly compared in Figure 14A. In the male animal four tissue/organ samples exceeded the relative Gb4Cer threshold value of ≥5% being spleen (10) reaching with 5.7% the top value, followed by kidney pelvis (19) with 5.6%, lung (14) with 5.2% and whole blood (24) with 5.1%. Tissues/organs of the ≤2.5% group were serum (25) with 2.0%, followed by cerebellum (23) with 1.8% and cerebrum (22) with 1.6% coming in last. Thus, the majority of eighteen sample values ranged in the interval between ≥2.5% and ≤5% indicating a moderate leveling of Gb4Cer expression together with less prominent peak values when compared to Gb3Cer expression (see Figure 13A). Finally, the cartoon of the swine (Figure 14B) emphasizes the tissue/organ distribution of the two piglets as a topographical atlas with circle diameters and “cake pieces” corresponding to Gb4Cer ratios. Since very similar tendencies were observed for tissues/organs of the female piglet, they are not further described in detail.

## 3. Discussion

A panel of 25 tissues and organs (including whole blood and serum) being implicated to a greater or lesser extent in the manifestation of the edema disease in weaned piglets, were scrutinized in this study in six-week-old weaned male and female piglets for the occurrence of the Stx2e receptors Gb3Cer, Gb4Cer and Forssman GSL. Stx2e has been shown in previous studies by others and us to bind to the globo-series GSLs Gb3Cer and Gb4Cer [25,26,27,38] and, as a unique feature for Stx2e among the other Stx subtypes analyzed so far, to the Forssman GSL (see explanations in Figure S1) [25]. Antibodies against Gb3Cer, Gb4Cer and Forssman GSL as well as Stx2e itself were employed in TLC overlay assays combined with mass spectrometry particularly for elucidation of variable receptor lipoforms.

All analyzed piglet tissues and organs of both genders were found positive for Gb3Cer and Gb4Cer, but considerably different regarding content of globo-series GSLs as demonstrated in this study. Only minor quantitative and slight qualitative differences were found for canonical Stx2e receptors Gb3Cer and Gb4Cer in the male and the female piglet of the same breed. In the male piglet, a somewhat higher content of the Stx2e receptors Gb3Cer and Gb4Cer counting for all organs and distinct hydroxylation of the ceramide lipid anchor confined to Gb3Cer of the male intestinal tract (duodenum, jejunum, ileum and colon) were observed. The presence of an additional hydroxyl group in the lipid moiety of a GSL seems important for the pathogenesis of bacterial infections and in particular for intestinal colonization of pathogens, since ceramide hydroxylation of GSL receptor molecules might play a functional role in increasing toxin affinity towards GSL receptors as shown for binding of Stxs to Gb3Cer [45]. Furthermore, hydroxylation of the ceramide moiety may exert a reinforcing effect for host-pathogen interaction suggesting an advantage for certain pathogens as shown for F4- and F6-fimbriated enterotoxigenic *E. coli* in adhesion studies with porcine intestinal GSLs [46,47] and F1C-fimbriated uropathogenic *E. coli* [48].

The Forssman GSL was detected in minute quantities only in GSL extracts of jejunum and quadriceps muscle of the male piglet being undetectable in all tissues/organs of the female piglet. Detection of trace quantities was solely verifiable with highly sensitive monoclonal antibody, but not by mass spectrometry due to extremely low quantities. However, this gender difference regarding restriction of Forssman GSL expression in the male piglet remains obscure and requires further in-depth investigations. Apart from that, Forssman GSL is expressed by various mammalian species (e.g. cat, mouse, rabbit and rat) [49], but seems to be absent in humans due to two mutations in the GBGT1 gene encoding the Forssman GSL synthetase [50] resulting in the lack of functioning α3GalNAc-transferase. Exceptions might be human individuals sharing the very rare and newly discovered enigmatic human histo-blood group system named FORS [51]. However, the situation remains confusing, because presence of Forssman GSL in sizeable quantities has been otherwise reported for normal human kidney [52].

Tissue distribution analysis of Gb3Cer and Gb4Cer has been performed in early investigations by Boyd and co-workers using weanling Yorkshire piglets [39]. TLC binding assays with VT2e (synonymous to Stx2e) revealed highest Gb3Cer concentrations in spleen and lung, followed by colon and kidney (medulla and cortex), whereas cerebrum and cerebellum were negative. This ranking is quite similar to our results using a different swine race (cross of German Large White and German Landrace mated to a Piétrain boar), indicating ubiquitous distribution of globo-series GSLs in the animals independent of the breed of pig. In contrast to the work of Boyd and collaborators [39] we were able to detect a minor content of Gb3Cer and Gb4Cer in cerebrum and cerebellum attributable to the use of highly sensitive polyclonal antibodies, whereas Stx2e also revealed negative results in our hands due to lower binding sensitivity of the toxin when compared to polyclonal anti-GSL antibodies. The exact lipoforms of Gb3Cer present in the various porcine tissues/organs, which have never been characterized in such detail before, are Gb3Cer species with ceramide moieties composed of sphingosine (d18:1) and a fatty acid with variable C16 up to C24 chain lengths. Gb3Cer variants of kidney cortex, medulla and pelvis with chiefly prevalence of Gb3Cer (d18:1, C24:1/C24:0) and Gb3Cer (d18:1, C22:0) over Gb3Cer (d18:1, C16:0), Gb3Cer (d18:1, C20:0) and Gb3Cer (d18:1, C18:0) (in this order) are typical for pig organs. The same holds true for the various Gb4Cer variants, exhibiting very similar ceramide variability as the Gb3Cer counterparts.

With regard to Gb4Cer (the prevalent Stx2e receptor), kidney medulla of Lingwood’s pigs [39] held position 2, colon ranged on place 6 and kidney cortex on position 10 in their sample collection. In our case, kidney medulla was found on place 18, colon on position 14 and kidney cortex on position 21 (results of the male animal). Both sets of results for these organs are not really plausible concerning their heavy involvement in the development of Stx2e-mediated porcine disease, although kidney pelvis ranked on top 2 position in our sample collection. Intravenously administered radiolabelled VT2e was retrieved mostly in red blood cells [39] what is in good agreement with our results when unraveling Gb4Cer in GSL extracts of whole blood using TLC immunodetection (rank 4) and TLC overlay assays using Stx2e. Stx2e gave uppermost intensive Gb4Cer positive bands suggesting that blood cells act as toxin carriers in edema disease [39], supported by binding studies of Stx2e with porcine erythrocytes [53] and identification of Stx-binding sites on porcine peripheral blood leukocytes [17]. Employing indirect immunofluorescence microscopy of thin sections of porcine intestine (jejunum, ileum and colon) and TLC overlay assay detection of corresponding total lipid extracts revealed presence of VT2e receptors Gb3Cer and Gb4Cer in porcine gut [2]. However, their role in absorption of VT2e was found to be unclear, because intraintestinal inoculation of pigs with large quantities of VT2e did not result in edema disease [2]. Interestingly, we found a high extent of hydroxylated ceramides in the male intestinal samples for Gb3Cer (but not for Gb4Cer), which might influence the accessibility of hydroxylated Gb3Cer species towards Stx2e. However, this is a highly speculative unproved interpretation based on preliminary results at this stage of research.

Further studies aimed at the localization of binding sites using VT2e for immunohistochemistry assays of selected pig tissues and organs revealed labeling throughout the tissue but variable labeling between and within blood vessels [40]. Anyway, endothelial and vascular smooth muscle cells of the eyelid were identified as the Stx2e receptor-positive cells being in agreement with our positive binding assays for Gb3Cer and Gb4Cer detected in eyelid GSL extracts. From the work of Waddell and collaborators [40], the other beforehand discussed reports and several other studies on porcine edema disease, it can be concluded that factors others than simply the presence of Stx2e receptor GSLs may influence the development of the classical symptoms and in particular severe tissue and organ lesions in edema disease.

Upon infection of a pig with Stx2e-producing STEC, the toxin is absorbed from the small intestine into the circulation and transported to the sensitive tissues, followed by manifestation of edema disease as vascular necrosis, edemas, neurologic signs, and death [3,54]. Edema disease seems to be chiefly a disease of the vasculature characterized by systemic vascular endothelial damage. This has been previously shown in vivo in pigs with edema disease that was produced by oral inoculation with Stx2e-producing *E. coli* [55]. Endothelial cell changes, for example of brain arterioles, ranged from acute swelling to necrosis and detachment from basement membranes, which could be attributed to the cytotoxic effects of Stx2e. Endothelial injury may result in breakdown of the blood–brain barrier and neurologic signs such as ataxia, incoordination and recumbency of susceptible swines [56]. In this context we have previously shown in an in vitro study that Gb3Cer- and Gb4Cer-expressing primary porcine brain capillary endothelial cells are targets for Stx2e and that Stx2e is capable to efficiently destroy the blood–brain barrier endothelium [38]. Interestingly, only very low amounts of Gb3Cer and Gb4Cer were detected by us in cerebrum and cerebellum. This finding suggests an extremely low tissue content of Stx2e receptors, because found globo-series GSLs may derive preferably from endothelial cells of highly vascularized cerebrum and cerebellum. Thus Stx2e-mediated crash of the blood–brain barrier might be the major reason of cerebral edemas with nervous disorders as main clinical signs [3].

Stx-mediated injury of pig or human target cells may depend in particular (but not only) on the cell- and/or organ-specific Stx GSL receptor repertoire. Different cellular cytotoxicity of the various Stx-subtypes probably relates to differences in the toxins’ receptor specificity: Stx1a and Stx2a (associated with hemorrhagic colitis and HUS in humans) bind preferentially to Gb3Cer, whereas Stx2e (associated with edema disease in pigs) prefers Gb4Cer [25,26]. Gb4Cer is present, although in different amounts, in all pig organs and tissues analyzed in this study, whereby a particularly strong interaction of Stx2e towards Gb4Cer in certain animal organs suggests their preponderant involvement in the onset of edema disease. Although it is tempting to speculate about such correlations, our data suggests that Stx-receptor expression alone does not satisfactorily explain the preferred targeting of Stxs of certain tissues and/organs and the concomitant outcome of an STEC infection. Furthermore, one should bear in mind that STEC produce a cocktail of virulence factors [57,58], which might act in a cumulative manner in the complex process of STEC infections.

Zoonotic potential of Stx2e-producing STEC strains has been suggested for humans by some occasional cases in the past, where Stx2e has been found being associated with severe human hemorrhagic colitis or HUS [59,60,61]. However, the rarity of detection of Stx2e-releasing STEC from human feces and mild course of disease could relate to lack of pathogenicity of Stx2e in humans [62,63]. Since Stx2e-producing STEC: (i) are present in pork products [5]; (ii) differ in their virulence profiles (depending on human or pig origin with potential to adopt to the specific host in which they may cause various forms of disease) [64]; and (iii) might be transmissible between humans and pigs [65], the possible zoonotic potential of Stx2e-producing STEC seems to threaten humans like the “Sword of Damocles”, although it never killed a human being.

Besides the putative zoonotic hazard of Stx2e-releasing STEC (the majority of STEC in pig feces) [66,67,68], swine represents a reservoir for clinically relevant STEC strains associated with human illness and pork products have been linked with outbreaks associated with human pathogenic STEC [5,69]. However, the relationship between STEC of swine origin and human illness has yet to be determined [5] and future in-depth investigations, e.g., employing novel antibodies against Stx2e for clinical applications [70,71], are required to clarify to which extent swine STEC are of public concern [68,72] and to further our knowledge on the Stx expression and distribution as well as its role in the environment [73].

## 4. Materials and Methods

### 4.1. Pig Tissues and Organs

Samples of 25 tissues and organs were prepared from a 6 weeks old healthy male and a healthy female piglet, which were obtained from a local slaughter. The parental sows were a cross of German Large White (Deutsches Edelschwein) and German Landrace (Deutsche Landrasse) mated to a Piétrain boar. The analyzed swine tissues and organs are listed in Table 1 and the definite wet weights of dissected samples are provided in Table S1.

### 4.2. Preparation of GSL Extracts

Tissues and organs were minced with a homogenizer (Polytron^®^ PT 1200 with tip PT-DA 1205/2, Kinematica AG, Littau, Switzerland) and extracted twice with 2 mL of chloroform/methanol (1/2, *v*/*v*), 2 mL of chloroform/methanol (1/1, *v*/*v*), and 2 mL of chloroform/methanol (2/1, *v*/*v*) (all purchased from Merck, Darmstadt, Germany). The supernatants of each extract were combined (12 mL), dried by rotary evaporation (Heidolph, Schwabach, Germany) and co-extracted phospholipids and triglycerides, which make up the major lipids in crude lipid extracts, were saponified with 4 mL of 1 N NaOH (Merck) solution for 1 h at 37 °C. Afterwards, the alkaline solution was neutralized dropwise with 400 µL of 10 N HCl (Merck), followed by dialysis against deionized water and drying by rotary evaporation. The extracts were dissolved in defined volumes of chloroform/methanol (2/1, *v*/*v*) corresponding to 0.1 mg wet weight per µL or to 0.1 µL of serum per µL dissolvent.

### 4.3. Antibodies and Stx2e

Polyclonal chicken IgY anti-Gb3Cer and anti-Gb4Cer antibodies with previously described specificities [42,74,75] and a monoclonal rat IgM anti-Forssman GSL antibody (clone IIC2), produced by Bethke and co-workers [41] and described as a highly specific tool for the detection of Forssman GSL [25,38,76], were employed for TLC overlay assays. Stx2e-containing supernatant of STEC strain S123G of serotype O139:K82 was used as previously described [25]. Mouse anti-Stx2 antibody (clone VT 135/6-B9, 2.75 mg/mL) was purchased from SIFIN GmbH (Berlin, Germany). Secondary alkaline phosphatase (AP)-conjugated affinity-purified polyclonal rabbit anti-chicken IgY (code 303-055-033), goat anti-rat IgG + IgM (code 112-055-044) and goat anti-mouse IgG (code 115-055-003) antibodies were from Dianova (Hamburg, Germany).

### 4.4. Reference Glycosphingolipids (GSLs)

A mixture of neutral GSLs composed of almost equimolar amounts of Gb3Cer, Gb4Cer and Forssman GSL was prepared as previously described [25] and used as positive control for TLC overlay assays. Major Gb3Cer and Gb4Cer reference species with a constant sphingosine (d18:1) moiety but variable fatty acyl chain are Gb3Cer lipoforms carrying C24:1/C24:0, C22:0 or C16:0 fatty acid with *m*/*z* values 1156.72/1158.74, 1130.75 or 1046.65, respectively, and the corresponding Gb4Cer variants with *m*/*z* 1359.75/1361.76, 1333.82 or 1249.77, respectively (see MS^1^ spectrum of Figure 5A in [25]). Major Forssman GSL variants carry Cer (d18:1, C24:1/C24:0) and Cer (d18:1, C22:0) lipid anchors with corresponding GSL *m*/*z* values of 1562.94/1564.95 and 1536.93, respectively (see MS^1^ spectrum of Figure 5B in [25]). The nomenclature of GSLs follows the IUPAC-IUB recommendations 1997 [77].

### 4.5. High-Performance Thin-Layer Chromatography (TLC) of GSLs

GSLs were applied to silica gel 60 precoated glass plates (HPTLC plates, size 10 cm × 10 cm, thickness 0.2 mm of the silica gel layer, no. 1.05633.0001; Merck) with an automatic sample applicator (Linomat 5, CAMAG, Muttenz, Switzerland). Applied GSL aliquots of tissues and organs correspond to 2 mg of wet weight each and that one of serum to 2 µL of volume. Separation of neutral GSLs was performed in chloroform/methanol/water (120/70/17, each by vol.) for 20 min. Separated GSLs were detected using TLC immunostaining (see next chapter). Immunostained bands were densitometrically quantified with a CD 60 scanner (Desaga, Heidelberg, Germany, software ProQuant^®^, version 1.06.000) in reflectance mode at a wavelength of 630 nm (indolyl phosphate) with light beam slit dimensions of 0.02 mm × 4 mm.

### 4.6. TLC Overlay Assay Detection of GSLs with Antibodies and Stx2e

After GSL separation and fixation of the silica gel with poly (isobutylmethacrylate) (Plexigum P28, Darmstadt, Germany), TLC immunostaining overlay assays were carried out with anti-Gb3Cer, anti-Gb4Cer and anti-Forssman GSL antibodies (see Section 4.3). The antibodies were diluted (1:2000, anti-Gb3Cer and anti-Gb4Cer antibodies; 1:20 hybridoma supernatant containing anti-Forssman antibody) with 1% (*w*/*v*) bovine serum albumin (BSA) (Serva, Heidelberg, Germany) in phosphate-buffered saline (PBS) (Lonza, Verviers, Belgium). The Stx2e-containing STEC supernatant was used undiluted. The anti-Stx2 antibody was applied in 1:1000 dilution and the secondary AP-conjugated antibodies were used as 1:2000 dilutions (both in 1% BSA in PBS) as previously described [25,43,44,78,79,80]. Bound secondary antibodies were detected with 0.05% (*w*/*v*) 5-bromo-4-chloro-3-indolyl phosphate *p*-toluidine salt (Roth, Karlsruhe, Germany) in glycine buffer (pH 10.4) (Serva), which generates a blue precipitate at sites of antibody binding on the TLC plate.

### 4.7. Extraction of GSLs from Silica Gel of Immunostained TLC Bands

Before extraction of GSLs out of the silica gel from immunopositive TLC bands, the poly(isobutyl methacrylate) fixative (Plexigum P28), which is required for silica gel fixation on the glass plate throughout the TLC overlay assays, has to be removed from the plate [81]. To this end, immunochromatograms were submerged three times in distilled chloroform. After evaporation of remnant chloroform from the plate in a fume hood, the immunopositive bands were scraped off and the silica gel was transferred into a small conical vial followed by threefold extraction of the GSL with 300 µL of methanol with intermediate centrifugations at 14,000× *g* (Eppendorf, Hamburg, Germany). The GSL-containing supernatants were pooled, gently dried under a stream of nitrogen and submitted to mass spectrometry measurements (for further details refer to [36]).

### 4.8. Mass Spectrometry

Electrospray ionization mass spectrometry (ESI-MS) was carried out with a quadrupole time-of-flight mass spectrometer endowed with a nanospray manipulator (Micromass, Manchester, UK) in the positive ion mode as described previously [25,38,44,79]. Dried GSL extracts from silica gel were dissolved in methanol containing 1% (*v*/*v*) formic acid (Sigma-Aldrich, Steinheim, Germany) and analyzed in the positive ion mode by ESI-MS^1^. For structural elucidation of selected species low-energy collision-induced dissociation (CID) was performed using argon as collision gas (MS^2^). The nomenclature introduced by Domon and Costello [82,83] was used for the assignment of the fragment ions obtained in MS^2^ analysis.

### 4.9. Statistics

The relative content of antibody-detected Gb3Cer or Gb4Cer in the porcine tissues and organs was determined in triplicate using the scan values of the TLC overlay assays. The intensities of immunostained Gb3Cer or Gb4Cer bands from 25 tissues/organs of an animal were summed up, normalized to 100%, and the percentage content of each sample was calculated from the corresponding relative peak intensities. The strength of association between Gb3Cer and Gb4Cer content in one and the same animal and between Gb3Cer and Gb4Cer amounts of the male and female animal, respectively, was assessed employing Spearman’s rank correlation test, i.e., calculating the rank correlation coefficient ρ of Spearman. Statistic was done in R software (version 3.2.0) [84]. Ranks were appointed to measurement values obtained by densitometric scanning of immunostained GSL bands, whereby rank 1 corresponds to the highest value, rank 2 to the second highest value, etc., and rank 25 to the lowest value. All tests were two-tailed and the ρ and the respective *p*-values were calculated with R software, whereby ρ-values were considered significant at *p*-values < 0.01 (significance level).

## Figures and Tables

**Table 1:** Ranked distribution of Stx2e receptors Gb3Cer and Gb4Cer in tissues and organs of male and female piglet.

	Gb3Cer				Gb4Cer				
Rank ^a^	No. ^b^	Male	No. ^c^	Female	No. ^b^	Male	No. ^c^	Female	
1	6	intestine jejunum	14	lung	10	spleen	10	spleen	**I ^d^**
2	14	lung	10	spleen	19	kidney pelvis	14	lung	
3	5	intestine duodenum	16	intestine colon	14	lung	9	stomach	
4	7	intestine ileum	7	intestine ileum	24	whole blood ^f^	24	whole blood ^f^	
5	16	intestine colon	15	lymph nodes ^e^	11	pancreas	19	kidney pelvis	
6	10	spleen	5	intestine duodenum	7	intestine ileum	13	gall bladder	
7	15	lymph nodes ^e^	6	intestine jejunum	3	nasal bridge	15	lymph nodes ^e^	
8	19	kidney pelvis	19	kidney pelvis	1	earlobe	11	pancreas	
9	20	urinary bladder	20	urinary bladder	9	stomach	7	intestine ileum	
10	21	heart	12	liver	8	ureter	12	liver	
11	3	nasal bridge	21	heart	21	heart	5	intestine duodenum	**II ^d^**
12	8	ureter	9	stomach	4	quadriceps muscle	21	heart	
13	18	kidney medulla	24	whole blood ^f^	15	Lymphnodes ^e^	6	intestine jejunum	
14	12	liver	17	kidney cortex	16	intestine colon	8	ureter	
15	4	quadriceps muscle	8	ureter	2	eyelid	2	eyelid	
16	2	eyelid	1	earlobe	5	intestine duodenum	16	intestine colon	
17	9	stomach	2	eyelid	6	intestine jejunum	20	urinary bladder	
18	11	pancreas	18	kidney medulla	13	gall bladder	1	earlobe	
19	13	gall bladder	11	pancreas	18	kidney medulla	3	nasal bridge	
20	24	whole blood ^f^	13	gall bladder	20	urinary bladder	18	kidney medulla	**III ^d^**
21	23	cerebellum	3	nasal bridge	17	kidney cortex	17	kidney cortex	
22	22	cerebrum	22	cerebrum	12	liver	4	quadriceps muscle	
23	17	kidney cortex	23	cerebellum	25	serum	23	cerebellum	
24	1	earlobe	4	quadriceps muscle	23	cerebellum	25	serum	
25	25	serum	25	serum	22	cerebrum	22	cerebrum	

^a^ Ranks were assigned to densitometric values of immunostained GSL bands, whereby rank 1 corresponds to the highest value, rank 2 to the second highest value, etc., and rank 25 to the lowest value; ^b^ Corresponding to identical sample numbering in Figure 1 and Figure 4 (male piglet); ^c^ Corresponding to identical sample numbering in Figure 8 and Figure 9 (female piglet); ^d^ Tissues/organs were synopsized in three categories according to their ranked Stx2e receptor quantities ranging from high (I) over moderate (II) to low content (III); ^e^ From small intestine; ^f^ EDTA-treated.

## Supplementary Materials

The following are available online at www.mdpi.com/2072-6651/8/12/357/s1, Figure S1: Stx receptors Gb3Cer, Gb4Cer and Forssman GSL, Figure S2: MS^2^ spectrum of Gb3Cer (d18:1, C16:0-OH) from male duodenum (**A**); and corresponding fragmentation scheme (**B**), Figure S3: MS^2^ spectrum of Gb3Cer (d18:1, C24:0-OH) from male cortex (**A**); and corresponding fragmentation scheme (**B**), Figure S4: Overview mass spectra of Gb3Cer lipoforms derived from: duodenum (**A**); and colon (**B**) of the female piglet, Figure S5: Overview mass spectra of Gb3Cer lipoforms derived from cortex (**A**); medulla (**B**); and pelvis (**C**) of the female piglet, Table S1: Synopsis of tissue/organ wet weights and ranks of Gb3Cer and Gb4Cer content of samples from male and female weaned piglet.
