# Supplementary material for: A Topographical Atlas of Shiga Toxin 2e Receptor Distribution in the Tissues of Weaned Piglets
Source: Toxins (Basel). 2016 Nov 30;8(12):357. doi: 10.3390/toxins8120357 (PMC5198551; doi:10.3390/toxins8120357)
Supplement: Supplementary file 1 [file toxins-08-00357-s001.pdf]

Supplementary Materials: A Topographical Atlas of Shiga Toxin 2e Receptor Distribution in the Tissues of Weaned Piglets

Daniel Steil, Robert Bonse, Iris Meisen, Gottfried Pohlentz, German Vallejo, Helge Karch and Johannes Müthing

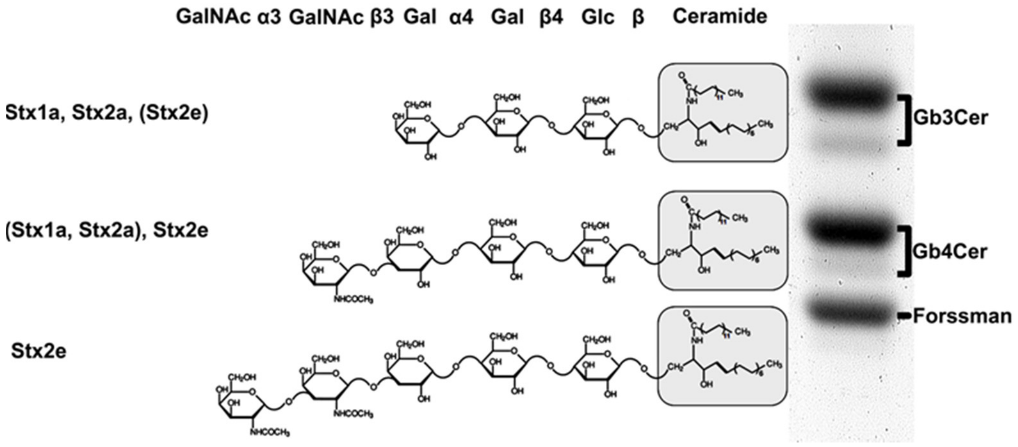

Figure S1. Stx receptors Gb3Cer, Gb4Cer and Forssman GSL. Structures of Gb3Cer, Gb4Cer and Forssman GSL (middle panel, from top to bottom) together with indications of preferential receptor recognition of Stx1a, Stx2a and Stx2e (left panel) and orcinol-stained thin-layer chromatogram of a GSL reference mixture containing the three receptor GSLs (right panel). Stx-subtypes in parentheses indicate less binding potential of respective Stx.

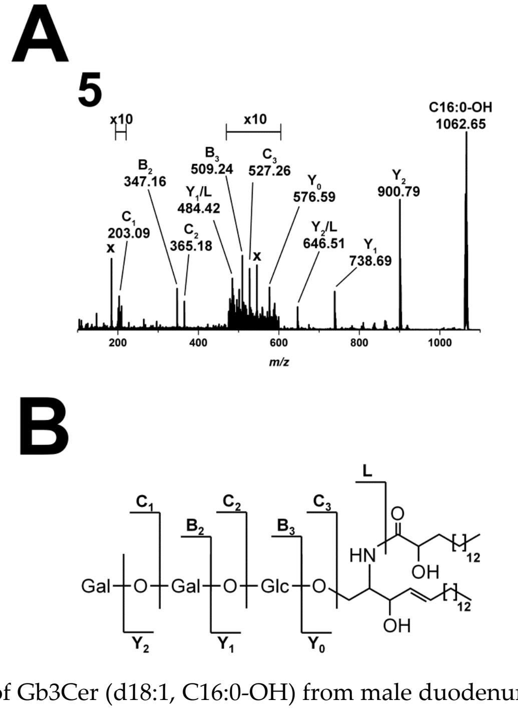

Figure S2. MS[2] spectrum of Gb3Cer (d18:1, C16:0-OH) from male duodenum (**A**) and corresponding fragmentation scheme (**B**). (**A**) Ions marked with an 'x' are not further characterized compounds; '5' corresponds to sample number 5 (intestine duodenum, see Table 1 and Table S1); (**B**) The fragment acronyms explain the fragment ions obtained by CID mass spectrometry from precursor ions at *m*/*z* 1062.65.

Table S1. Synopsis of tissue/organ wet weights and ranks of Gb3Cer and Gb4Cer content of samples from male and female weaned piglet.

No. [a]	Tissue/Organ	Wet Weight (mg)		Rank Gb3Cer		Rank Gb4Cer	
		Male	Female	Male	Female	Male	Female
1	earlobe	189.5	511.1	24	16	8	18
2	eyelid	240.5	262.6	16	17	15	15
3	nasal bridge	80.8	302.3	11	21	7	19
4	quadriceps muscle	314.8	804.9	15	24	12	22
5	intestine duodenum	449.1	450.9	3	6	16	11
6	intestine jejunum	141.7	375.7	1	7	17	13
7	intestine ileum	234.3	378.5	4	4	6	9
8	ureter	118.5	140.8	12	15	10	14
9	stomach	424.7	364	17	12	9	3
10	spleen	173.5	352.6	6	2	1	1
11	pancreas	248.3	871.1	18	19	5	8
12	liver	501.6	627.4	14	10	22	10
13	gall bladder	101.4	234.5	19	20	18	6
14	lung	279.2	464.1	2	1	3	2
15	lymph nodes [b]	108.3	588.2	7	5	13	7
16	intestine colon	223.4	564.1	5	3	14	16
17	kidney cortex	162.1	473.4	23	14	21	21
18	kidney medulla	36.4	57.4	13	18	19	20
19	kidney pelvis	123.2	149.1	8	8	2	5
20	urinary bladder	261.5	320.7	9	9	20	17
21	heart	412.4	617.9	10	11	11	12
22	cerebrum	612.7	768	22	22	25	25
23	cerebellum	308	928	21	23	24	23
24	whole blood	961.3	615.1	20	13	4	4
25	serum [c]	500	500	25	25	23	24

[a] Corresponding to identical sample numbering in Figures 1 and 4 (male piglet) and Figures 8 and 9 (female piglet) as well as in Table 1; [b] From small intestine; [c] Volume in microliter (μL).

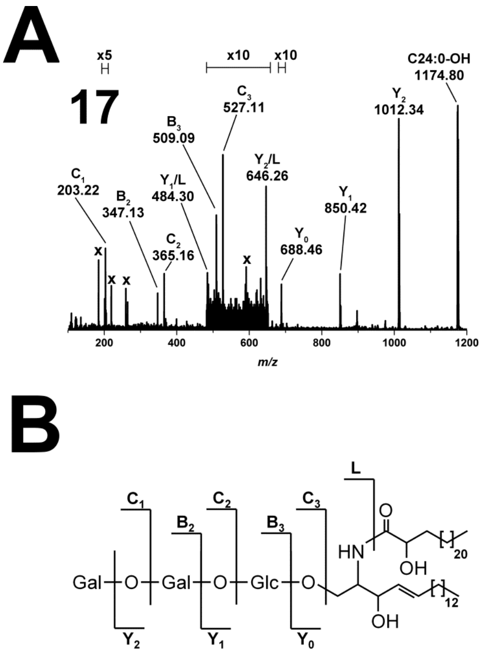

Figure S3. MS² spectrum of Gb3Cer (d18:1, C24:0-OH) from male cortex (**A**) and corresponding fragmentation scheme (**B**). (**A**) Ions marked with an 'x' are not further characterized compounds; '17' corresponds to sample number 17 (kidney cortex, see Table 1 and Table S1); (**B**) The fragment acronyms explain the fragment ions obtained by CID mass spectrometry from precursor ions at *m*/*z* 1174.80.

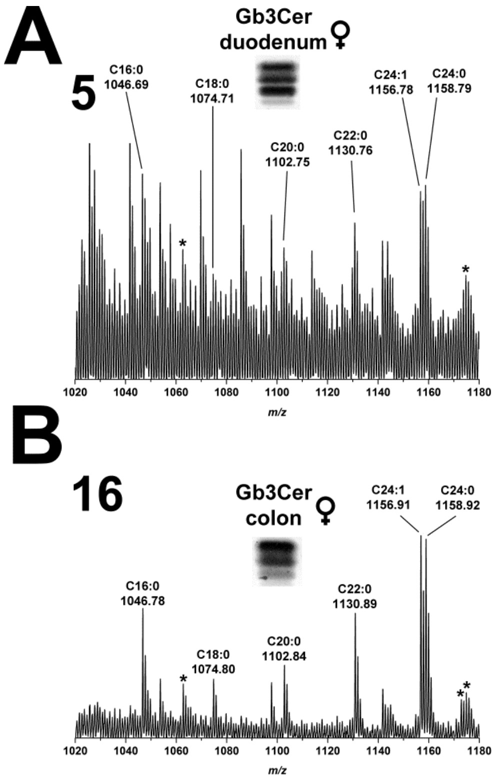

Figure S4. Overview mass spectra of Gb3Cer lipoforms derived from duodenum (**A**) and colon (**B**) of the female piglet. The MS[1] spectra display the *m*/*z* range between 1020 and 1180 comprising Gb3Cer lipoforms with variable fatty acid from C16:0 up to C24:1/C24:0 as assigned in the spectrum. All Gb3Cer variants carry constant sphingosine (d18:1) in their respective ceramide moiety. The asterisks point to mass shifts of 15.99 u indicating presence of an additional OH-group in the ceramide moiety, most likely linked to the fatty acyl chain, in comparison to non-hydroxylated species. That are Gb3Cer variants of the female duodenum ((**A**) sample no. 5, see Figure 1, Table 1 and Table S1) with C16:0-OH (*m*/*z* 1062.70) and C24:0-OH (*m*/*z* 1174.78). Identified hydroxylated Gb3Cer variants of the female colon ((**B**) sample 16, see Figure 1, Table 1 and Table S1) are lipoforms with C16:0-OH (*m*/*z* 1062.78) and C24:1-OH/C24:0-OH (*m*/*z* 1172.89/1174.87).

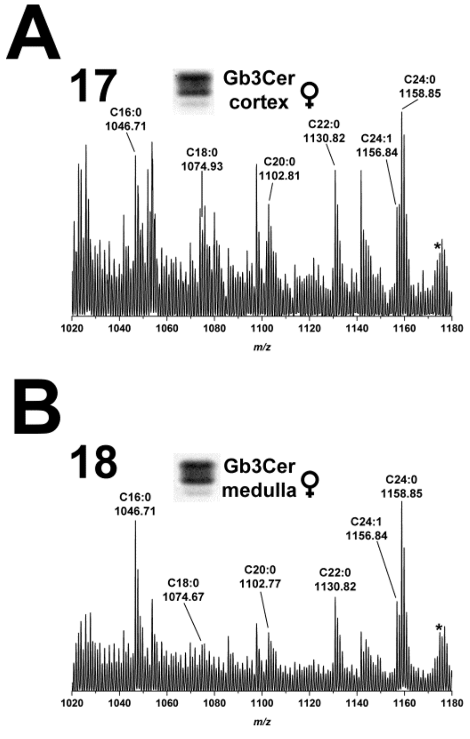

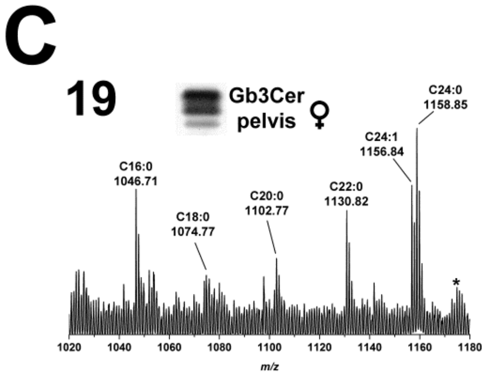

Figure S5. Overview mass spectra of Gb3Cer lipoforms derived from cortex (**A**); medulla (**B**) and pelvis (**C**) of the female piglet. The MS^1 spectra depict the *m*/*z* range from 1020 to 1180 encompassing Gb3Cer variants with variable fatty acid from C16:0 up to C24:1/C24:0 as marked in the spectrum. All Gb3Cer variants harbor constant sphingosine (d18:1) in their respective ceramide moiety. The asterisks indicate hydroxylation of the ceramide portion, most likely of the fatty acid, of Gb3Cer with C24:0-OH (*m*/*z* 1174.84) in the female cortex ((**A**) sample no. 17); female medulla ((**B**) sample no. 18), and female pelvis ((**C**) sample no. 19) (see Figure 1, Table 1, and Table S1).
